# Exploring associations between ADHD symptoms and emotional problems from childhood to adulthood: shared aetiology or possible causal relationship?

**DOI:** 10.1017/S0033291724002514

**Published:** 2024-11

**Authors:** Yuan You, Olakunle A. Oginni, Fruhling V. Rijsdijk, Kai X. Lim, Helena M. S. Zavos, Tom A. McAdams

**Affiliations:** 1 Social, Genetic and Developmental Psychiatry Centre, Institute of Psychiatry, Psychology & Neuroscience, King's College London, London, UK; 2 Department of Mental Health, Obafemi Awolowo University, Ile-Ife, Nigeria; 3 Department of Psychology, Faculty of Social Sciences, Anton de Kom University, Paramaribo, Suriname; 4 Department of Psychology, Institute of Psychiatry, Psychology & Neuroscience, Kings College London, London, UK; 5 Promenta Centre, University of Oslo, Norway

**Keywords:** attention deficit hyperactivity disorder, direction of causation, emotional problems, TEDS, twin study

## Abstract

**Background:**

ADHD symptoms are associated with emotional problems such as depressive and anxiety symptoms from early childhood to adulthood, with the association increasing with age. A shared aetiology and/or a causal relationship could explain their correlation. In the current study, we explore these explanations for the association between ADHD symptoms and emotional problems from childhood to adulthood.

**Methods:**

Data were drawn from the Twins Early Development Study (TEDS), including 3675 identical and 7063 non-identical twin pairs. ADHD symptoms and emotional symptoms were reported by parents from childhood to adulthood. Self-report scales were included from early adolescence. Five direction of causation (DoC) twin models were fitted to distinguish whether associations were better explained by shared aetiology and/or causal relationships in early childhood, mid-childhood, early adolescence, late adolescence, and early adulthood. Follow-up analyses explored associations for the two subdomains of ADHD symptoms, hyperactivity-impulsivity and inattention, separately.

**Results:**

The association between ADHD symptoms and emotional problems increased in magnitude from early childhood to adulthood. In the best-fitting models, positive genetic overlap played an important role in this association at all stages. A negative causal effect running from ADHD symptoms to emotional problems was also detected in early childhood and mid-childhood. When distinguishing ADHD subdomains, the apparent protective effect of ADHD symptoms on emotional problems in childhood was mostly driven by hyperactivity-impulsivity.

**Conclusions:**

Genetic overlap plays an important role in the association between ADHD symptoms and emotional problems. Hyperactivity-impulsivity may protect children from emotional problems in childhood, but this protective effect diminishes after adolescence.

## Introduction

Developmental continuity of ADHD symptoms is common (Biederman et al., 1998; Cak et al., 2013). The rates and types of ADHD comorbidities can however change across the developmental course. For example, both ADHD symptoms and clinically diagnosed ADHD are associated with conduct problems and neurodevelopmental issues in childhood, while evidence suggests that emotional problems become more associated with ADHD symptoms with age (e.g. Farhat et al., 2022; Franke et al., 2018; Sadek, 2018; Thapar, Harrington, & McGuffin, 2001). Up to 33.5% of children and adolescents with ADHD also have anxiety disorder, increasing to 50% in adulthood (D'Agati, Curatolo, & Mazzone, 2019; Sadek, 2016, 2018). These increasing associations have also been found at the subclinical level, with ADHD symptoms (especially inattention) in adulthood being associated with emotional problems (e.g. Jarrett, 2016; Leikauf & Solanto, 2017; Stanton & Watson, 2016).

Several studies have examined the aetiology underlying the covariance between ADHD symptoms and emotional problems. Genetic overlap has been found to account for 20% to 60% of the association between ADHD symptoms and emotional problems in twin studies (Brooker et al., 2020; Chen et al., 2016; Gustavson et al., 2021; Michelini, Eley, Gregory, & McAdams, 2015; Stern et al., 2020); and in genomic studies, risk alleles associated with ADHD have been found to increase risk for emotional problems (The Brainstorm Consortium et al., 2018; Brikell et al., 2020; Riglin et al., 2021). However, these studies have not considered the mechanisms that underlie the genetic correlation between ADHD symptoms and emotional problems. The common interpretation is that a genetic correlation indexes a common cause: a form of horizontal pleiotropy, whereby the same genetic factors influence both ADHD and emotional problems, thus explaining their genetic covariance. An alternative explanation involves causal influence between phenotypes: a form of vertical pleiotropy, whereby ADHD causally influences emotional problems (and/or vice versa), so genetic factors influencing ADHD influence emotional problems via this causal process, thus explaining their genetic covariance. To date, the majority of models used to estimate genetic covariance between ADHD symptoms and emotional problems have not been designed to distinguish horizontal from vertical pleiotropy.

It could be the case that ADHD symptoms cause emotional problems (such as depression and anxiety), and that this effect increases in magnitude as children age, thus explaining their increasing rate of co-occurrence. Such an increasing causal effect could occur if the norms related to ADHD-linked behaviors change over time. For example, if hyperactive behaviour becomes less acceptable over time, and leads to negative consequences, it may become increasingly linked with emotional problems. Externalizing behaviors such as aggression are known to lead to more negative consequences for older children (Dodge, 1980; Racz, Putnick, Suwalsky, Hendricks, & Bornstein, 2017). As children get older, there is also an increased expectation that they are able to focus in school. Inattention, therefore, may have a greater adverse impact on academic performance in adolescence than in younger children (Kawabata, Tseng, & Gau, 2012). Early adulthood is another important transition. In emerging adulthood, occupational and social impairment become important concerns for individuals with ADHD (LaCount, Hartung, Canu, & Knouse, 2019). As academic and occupational outcomes become increasingly salient during development, failure to achieve them satisfactorily may increase the likelihood of emotional problems over time. Overall, the increasing challenges children may meet as they grow older could lead to an increasing causal effect running from ADHD symptoms to emotional problems.

When considering the underlying mechanism between ADHD symptoms and emotional problems, it is important to consider that ADHD is comprised of two symptom domains: inattention and hyperactivity-impulsivity. Only moderate genetic overlap exists between domains (Greven, Rijsdijk, & Plomin, 2011; Sherman, Iacono, & McGue, 1997). Although heritability is high for both dimensions, the influence of dominant genetic effects is larger for inattention than hyperactivity-impulsivity, with additive genetic effects having a larger impact on the latter (Nikolas & Burt, 2010). The stability of the two ADHD subdomains also varies. Hyperactivity-impulsivity symptoms have been shown to become less prevalent with age whereas inattention symptoms are more stable from childhood to early adulthood (Döpfner, Hautmann, Görtz-Dorten, Klasen, & Ravens-Sieberer, 2015; Larsson, Dilshad, Lichtenstein, & Barker, 2011). Given these differences, it is possible that correlates of ADHD symptoms may differ in their associations with inattention *v.* hyperactivity-impulsivity, both in magnitude and underlying mechanism (Ghirardi et al., 2019; Plourde, Boivin, Brendgen, Vitaro, & Dionne, 2017). Results regarding the association between ADHD subdomains and emotional problems are mixed. One study showed that the association between anxiety and hyperactivity-impulsivity is lower than that between anxiety and inattention, about 0.35 and 0.03 separately (Michelini et al., 2015). However, another study reported that the comorbidity between ADHD symptoms and emotional problems may be specific to hyperactivity-impulsivity (Stern et al., 2020). These contrasting results might be due to differences in ages of the participants in each study (17 years and 5 years old respectively). Considering the subdomains of ADHD when exploring the association between ADHD symptoms and emotional problems across different developmental stages is therefore important.

In summary, the overlap between ADHD symptoms and emotional problems has been reported as largely genetic, with the association increasing in magnitude with age. However, no study to date has examined the overlap from childhood to adulthood within a single sample, and no study has compared models that test for shared aetiology *v.* causal influence as explanations for the genetic correlation between ADHD symptoms and emotional problems.

### Current study

In the current study, we explored associations between emotional problems and ADHD symptoms from early childhood to early adulthood using a longitudinal, population-based twin cohort. We fitted direction of causation twin models (Heath et al., 1993) to assess whether traits causally influenced one another and/or shared a common etiology at five developmental stages: early childhood (2–4 years old), mid-childhood (7–9 years old), early adolescence (12 years old), late adolescence (16 years old), and early adulthood (21 years old). We then repeated all analyses distinguishing the two symptom domains of ADHD (inattention and hyperactivity-impulsivity).

## Methods

### Sample

We used data from the Twins Early Development Study (TEDS), an ongoing longitudinal twin study on cognitive and behavioral development from childhood to adulthood (Rimfeld et al., 2019; Trouton, Spinath, & Plomin, 2002). Participants were selected from twins born in England and Wales between 1994 and 1996. Over 15 000 pairs of twins originally participated in the study and more than 10 000 pairs remain involved currently (Haworth, Davis, & Plomin, 2013; Rimfeld et al., 2019). In the initial screening procedure, twins with serious medical conditions were excluded. Overall, 3675 identical twin pairs and 7063 non-identical twin pairs were included in the current sample. 50.6% of the whole sample were females. In the early childhood stage, a total of 9617 pairs of twins provided data on ADHD or emotional problems. In the mid-childhood, early adolescence, late adolescence, and early adulthood stages, 8323 pairs, 5752 pairs, 4990 pairs, and 6495 pairs of twins respectively provided data on ADHD or emotional problems.

### Measures

Multiple measures of ADHD symptoms and emotional problems were used at the various developmental stages covered in our study. These measures are shown in Table 1.

**Table 1.** Measures in every stage

Symptoms	Reporters	Early childhood 2 to 4	Mid-childhood 7 to 9	Early adolescence 12	Late adolescence 16	Early adulthood 21
ADHD	Parent-report	Behar^a^ (ages 2, 3, 4)	Conners (age 8)	SDQ^b^ (age 12)	SDQ (age 16)	SDQ (age 21)
		SDQ (age 4)	SDQ (age 7, 9)	Conners^c^ (age 12)	Conners (age 16)	Conners (age 21)
	Child-report			SDQ(age 12)	SDQ(age 16)	Conners(age 21)
						SDQ(age 21)
Emotional problems	Parent-report	Behar (age 2, 3, 4)	DSM-IV criteria^d^ (age 7, 9)	SMFQ^e^ (age 12)	SMFQ (age 16)	SDQ (age 21)
		SDQ (age 4)	SDQ (age 7, 9)	SDQ (age 12)	ARBQ^f^ (age 16)	
	Child-report			SMFQ (age 12)	SMFQ (age 16)	SMFQ (age 21)
				SDQ (age 12)	SDQ (age 16)	SDQ (age 21)
						GA^g^ (age 21)

a Preschool Behavior Questionnaire (Behar; Behar and Stringfield, 1974).

b Strengths and Difficulties Questionnaire (SDQ; Goodman, 1997).

c Conners' Rating Scales-Revised (Conners; Conners, 2003).

d DSM-IV criteria for anxiety/depression disorders (APA, 1994).

e Short Mood and Feeling Questionnaire (SMFQ; Angold et al., 1995).

f Anxiety-Related Behaviors Questionnaire (ARBQ; Eley et al., 2003).

g Severity measure for generalized anxiety disorder – adult (GA; Craske et al., 2013).

### ADHD symptoms

In early childhood, ADHD symptoms were measured using the parent-report Preschool Behavior Questionnaire (Behar; Behar & Stringfield, 1974) at 2, 3, and 4 years old and the Strengths and Difficulties Questionnaire (SDQ; Goodman, 1997) at 4 years old. Cronbach's *α* was 0.71, 0.73, 0.73 for Behar and 0.76 for SDQ respectively. In mid-childhood, the parent-report SDQ was used at 7 and 9 years old and the Conners' Rating Scales-Revised (Conners; Conners, 2003) used at 8 years old. Cronbach's *α* was 0.76, 0.77 for SDQ and 0.92 for Conners. From early adolescence to early adulthood, we used both parent-report and child self-report symptoms including the SDQ and Conners, with Cronbach's *α*s of 0.76, 0.72, 0.71 for parent-report SDQ and 0.70, 0.73, 0.67 for self-report SDQ at 12, 16 and 21 years old. For Conners, the Cronbach's *α*s ranged from 0.89 to 0.92 for parent-report and self-report scales.

### Emotional problems

During early childhood, the parent-reported Behar and SDQ were used to measure emotional problems (Behar, 1977; Goodman, 1997). Cronbach's *α*s were 0.51, 0.58, and 0.58 for Behar at 2, 3, and 4 years old respectively, and 0.59 for SDQ at 4 years old. During mid-childhood, the parent-report SDQ and DSM-IV criteria for anxiety/depression disorders (APA, 1994) were used to measure emotional problems in children. Cronbach's *α*s were 0.59, 0.68 for SDQ, and 0.77, 0.67 for DSM-IV criteria at 7 and 9 years old, respectively. From early adolescence, parents and twins both reported on twins' emotional problems, using the SDQ and Short Mood and Feeling Questionnaire (SMFQ; Angold et al., 1995). Cronbach's *α*s were respectively 0.68, and 0.76 for parent-report SDQ at 12 and 21 years old, and 0.69, 0.70, 0.79 for child-report SDQ at 12, 16, and 21 years old. For the SMFQ, Cronbach's *α* was from 0.84 to 0.87 for both parent-report and self-report scales. In late adolescence, the parent-report Anxiety-Related Behaviors Questionnaire (ARBQ; Eley et al., 2003) was also used. And in early adulthood the self-report severity measure for generalized anxiety disorder – adult (GA; Craske et al., 2013) was used as well. Cronbach's *α*s for the ARBQ and the GA were 0.86 and 0.92, respectively.

### Genetic analysis

#### Twin models

All genetic analyses were conducted with the structural equation modelling R package (version 3.6.1) OpenMx (version 2.15.5; Neale et al., 2016). Additive genetic (A; *r*A = 1.0 for MZ twins and *r*A = 0.5 for DZ pairs), non-additive genetic (D; *r*D = 1.0 for MZ twins and *r*D = 0.25 for DZ pairs), shared environmental (C; *r*C = 1 for MZ and DZ twins), and nonshared environmental (E) variance components of traits were calculated using structural equation models. If *r*DZ > 1/2 *r*MZ, shared environmental components are suggested in the model, which contribute to higher similarity in DZ twins. Non-additive genetic components are indicated when *rM*Z > 2*rD*Z, (Boomsma, Busjahn, & Peltonen, 2002; Knopik, Neiderhiser, DeFries, & Plomin, 2017). It is not possible to estimate D components and C components at the same time in the classical twin design.

We calculated the relative importance of A, C/D, and E on all traits and determined whether there were dominant genetic effects besides additive genetic effects in ADHD symptoms and emotional problems. Previously, negative sibling interaction effects have been reported for parent-reported child ADHD symptoms, whereby parents ‘contrast’ their twin children with one another (Merwood et al., 2014; Rietveld, Posthuma, & Dolan, 2003). This reduces twin covariance, thus biasing model estimates. Based on previous work on this topic (Rietveld et al., 2003), we included sibling interaction terms in our models to account for potential contrast effects wherever the variances of DZ twins were greater than MZ twins.

#### Direction of causation models

We used Direction of causation (DoC) models (see online Supplementary Fig. S1) to explore whether associations between ADHD symptoms and emotional problems are causal. In the DoC models, differences in the aetiological structures of variables are required to determine the direction of any potential causal influence underlying an association between two variables (Heath et al., 1993; McAdams, Rijsdijk, Zavos, & Pingault, 2021; Verhulst & Estabrook, 2012). The aetiological structure of the covariance can then indicate which trait drives the cross-twin cross-trait correlation (Verhulst & Estabrook, 2012).

Measurement error can bias parameters in DoC models (Duffy & Martin, 1994; Heath et al., 1993). One way to circumvent this problem is to use common latent factors to index phenotypes instead of summary scales. When specifying latent factors with scales or items as indicators, the measurement error in each component (scale or item) is not included in the common factor. Such latent factors can therefore be considered free of measurement error and thus appropriate for use in DoC models (Verhulst & Estabrook, 2012). Therefore, in current study, we ran DoC models with ADHD symptoms and emotional problems specified as latent factors. Specifically, several scales were specified to load onto a single latent factor.

Previous studies indicate that emotional problems are usually influenced by additive genetic (A), shared environment (C), and nonshared environment variances (E) (Lamb et al., 2010), while ADHD symptoms are usually decomposed into additive genetic (A), dominant genetic (D), and nonshared environment variances (E; Rietveld et al., 2003). These etiological differences mean that it is possible to use a DoC model to assess potential causal relationships underlying covariance between ADHD symptoms and emotional problems.

**Figure 1.**
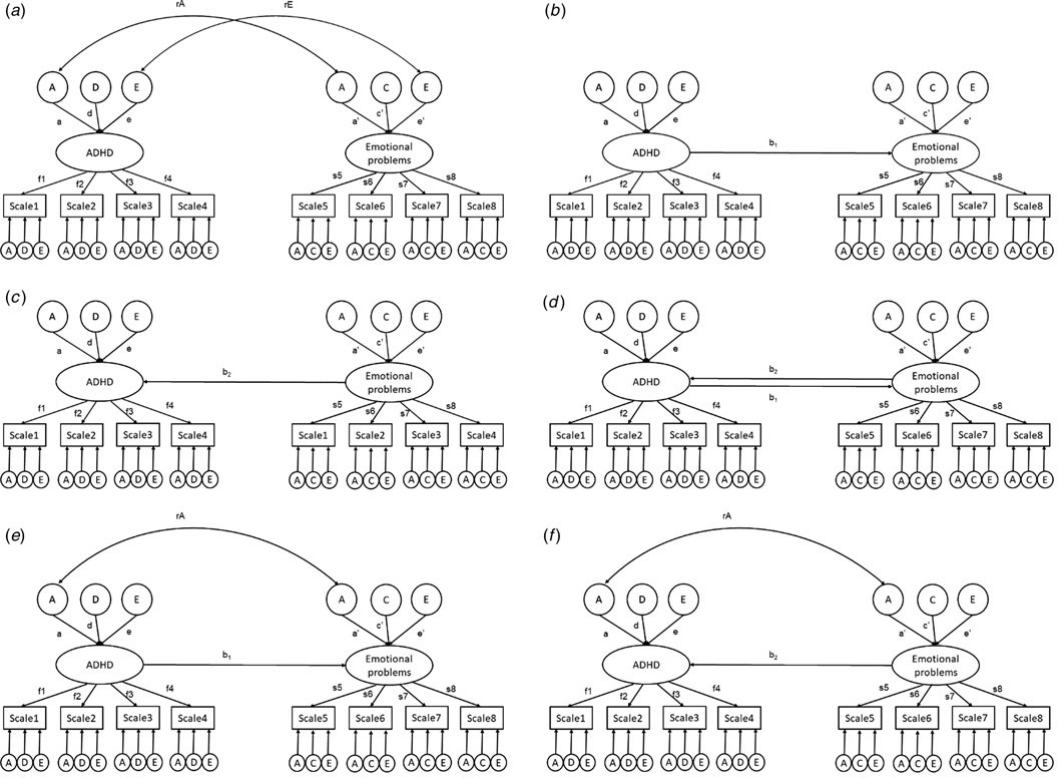
Modelling correlation and causality between ADHD and emotional problems. Bivariate etiological correlation model (a); unidirectional causation model (b and c); reciprocal causation model (d); hybrid causal-correlation model with both genetic correlations and causal paths (e & f). A, additive genetic effects; C, shared environmental effects; D, dominant genetic effects; E, nonshared environmental effects. *b*_1_, causal paths from ADHD to emotional problems; b_2_, causal paths from emotional problems to ADHD. *f*_1_; *f*_2_; *f*_3_; *f*_4_; *s*_5_; *s*_6_; *s*_7_; *s*_8_, factor loadings from the latent true scores or common pathways to the observed variables. *r*A, genetic correlations between the two traits. *r*E, specific environmental correlation between the two traits. a; d; c; e, additive genetic, dominant genetic, shared environmental and specific environmental impact on ADHD or emotional problems. Scales 1–4; Scale 5–8, the different scales used to assess ADHD and emotional problems at different time points.

In Fig. 1, we illustrate the series of models we fitted to our data to explain the covariance between ADHD symptoms and emotional problems: Bivariate etiological correlation models (see model (a) in online Supplementary Fig. S1) decompose the phenotypic association into genetic and environmental correlations. In these models, correlations between ADHD symptoms and emotional problems operate through the common latent influences, indicating shared etiology. Unidirectional causation models (see model (b) and model (c) in online Supplementary Fig. S1) are nested within the bivariate etiological correlation models (Medland & Hatemi, 2009; Verhulst & Estabrook, 2012), and specify covariance between traits as a causal effect of one trait on the other (or vice versa). In the unidirectional causation models, the genetic overlap between ADHD symptoms and emotional problems is totally explained by the causal effect of one trait on another. Reciprocal causation models whereby the covariance between two traits is explained by bidirectional causal effects are theoretically plausible (see model (d) in online Supplementary Fig. S1). However, the twin covariance structure between ADHD symptoms and emotional problems can not include evidence for both dominant genetic influences (from ADHD) and shared environment influences (from emotional problems) because the former requires that rMZ > 2rDZ and the latter requires that rMZ < 2rDZ. We therefore did not estimate reciprocal causation models but include one in the figure for completeness. Models that simultaneously estimate genetic correlations and causal paths (see model (e) and model (f) in online Supplementary Fig. S1) were fitted to the data as well.

The fit indices of the bivariate etiological correlation models, direction of causation models and hybrid causal-correlation models were compared to determine the likely mechanism underlying the association between the two traits.

#### The model fitting process

Structural equation models were fitted in OpenMx (version 2.15.5) using full-information maximum likelihood (FIML). We regressed out the effects of child gender and age before model fitting to control for their possible influences as covariates.

We provide several indices of overall model fit for each model we fitted: the Root Mean Square Error of Approximation (RMSEA; Steiger & Lind, 1980), the Tucker-Lewis fit index (TFI; Tucker & Lewis, 1973), and the Comparative Fit Index (CFI; Bentler, 1990). Nested models were compared using the −2 log-likelihood statistic (−2LL). A smaller −2LL indicates better fit to the data compared to models with larger −2LLs; Chi-square tests were then carried out to test whether −2LL differences were statistically significant. Akaike information criterion (AIC) and weights (Akaike, 1973, 1974) were used to compare the fit of non-nested models. Greater AIC differences provide stronger evidence for the model with the lower AIC, with differences of 0–2, 4–7, and 10 indicating no difference, some support and greater support, respectively (Burnham & Anderson, 2004). AICs can also be transformed into AIC weights, which indicate the conditional probabilities of a certain model being the best fit among a set of models (Wagenmakers & Farrell, 2004). Combining all these criteria, we determined which model best reflected the data.

We specified all the models at each timepoint to explore the associations between ADHD symptoms and emotional problems in early childhood (2–4 years old), mid-childhood (from 7 to 9 years old), early adolescence (12 years old), late adolescence (16 years old), and early adulthood (21 years old). We then re-ran the analyses focusing on the two ADHD subdomains. We were interested in the independent associations each ADHD domain had with emotional problems (e.g. the association between hyperactivity and emotional problems independent of inattention). Thus, in subdomain analyses, we controlled for e.g. inattention by regressing hyperactivity-impulsivity on inattention, saving the residual of this regression. We then used this residual in the subsequent analysis. In this manner, we intended to learn about the association between hyperactivity-impulsivity and emotional problems independent of inattention. By taking the reverse approach we learned about the association between inattention and emotional problems independent of hyperactivity-impulsivity.

## Results

### Phenotypic results

**Figure 2.**
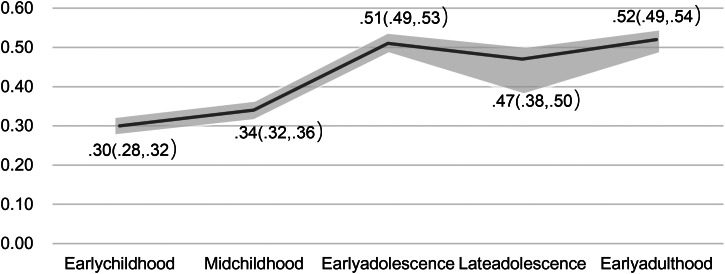
Phenotypic correlation between ADHD and emotional problems from early childhood to early adulthood.

**Figure 3.**
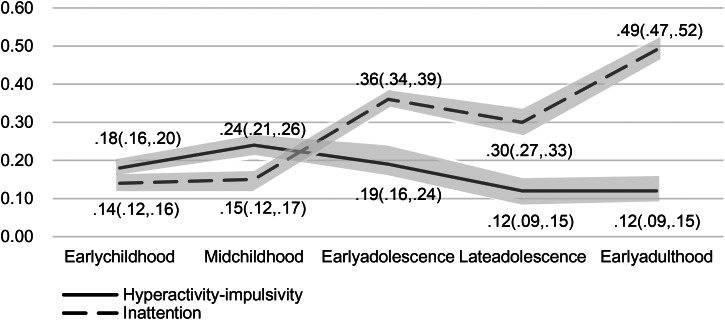
Phenotypic correlation between hyperactivity, inattention and emotional problems from early childhood to early adulthood.

Phenotypic correlations between latent factors indexing ADHD symptoms and emotional problems are presented in Figs 2 and 3. Based on the results, the highest correlation between ADHD and emotional problems was observed in early adulthood (*r* = 0.52, 95% confidence interval [CI] 0.49–0.54), while the lowest correlation was found in early childhood (*r* = 0.30, 95% CI 0.28–0.32). There was a significant increase in the correlation from mid-childhood (*r* = 0.34, 95% CI 0.32–0.36) to early adolescence (*r* = 0.51, 95% CI 0.49–0.53), with no overlap in the CIs. When considering the ADHD subdomains separately (whilst controlling for the other subdomain), the association between inattention and emotional problems most mirrored that of ADHD symptoms and emotional problems, increasing from early childhood (*r* = 0.14, 95% CI 0.12–0.16) to early adulthood (*r* = 0.49, 95% CI 0.47–0.52). Notably, the increase from mid-childhood (*r* = 0.15, 95% CI 0.12–0.17) to early adolescence (*r* = 0.36, 95% CI 0.34–0.39) and the increase from late adolescence (*r* = 0.30, 95% CI 0.27–0.33) to early adulthood (*r* = 0.49, 95% CI 0.47–0.52) were significant, with no overlap in the CIs. However, the correlation between hyperactivity-impulsivity and emotional problems increased from early childhood (*r* = 0.18, 95% CI 0.16–0.20), reached its highest level in mid-childhood (*r* = 0.24, 95% CI 0.21–0.26), and then declined from early adolescence (*r* = 0.19, 95% CI 0.16–0.24) to early adulthood (*r* = 0.12, 95% CI 0.09–0.15).

Overall, the association between hyperactivity-impulsivity and emotional problems (*r* = 0.24, 95% CI 0.21–0.26) was higher than that between inattention and emotional problems (*r* = 0.15, 95% CI 0.12–0.17) during mid-childhood. However, from early adolescence to early adulthood, the association between inattention and emotional problems (*r* = 0.36, 95% CI 0.34–0.39; *r* = 0.30, 95% CI 0.27–0.33; *r* = 0.49, 95% CI 0.47–0.52) was higher than that between hyperactivity-impulsivity and emotional problems (*r* = 0.19, 95% CI 0.16–0.24; *r* = 0.12, 95% CI 0.09–0.15; *r* = 0.12, 95% CI 0.09–0.15).

Attrition is a feature of all longitudinal studies. Because our analyses were focused on longitudinal changes in associations, we tested whether attrition was likely to have an impact on estimates of association. We did this by re-estimating early childhood correlations (our sample was most complete in early childhood) between ADHD symptoms and emotional problems in the subsample who continued to take part in mid-childhood, then again in the subsample who continued to early adolescence, then late adolescence and finally only in those who remained in the study until early adulthood. Results are shown in online Supplementary Table S1 and demonstrate that attrition did not affect the magnitude of associations.

#### Genetic results

As is shown in online Supplementary Table S2, in most measures of emotional problems, the correlations of DZ twins (*r*_DZ_ = 0.12–0.56) were close to or over half the magnitude of the MZ correlations (*r*_MZ_ = 0.33–0.83), indicating the potential presence of shared environmental influence as well as additive genetic influence and nonshared environmental influences. We therefore fitted ACE models for emotional problems across the different timepoints. Most of our models indicated a good fit to the data with RMSEA < 0.08, CFI > 0.9, and TFI > 0.9. The remaining models' CFI and TFI values were only slightly lower than the standards for good fit, with all TFI and CFI > 0.80.

For both parent-report and child-report ADHD symptoms, most MZ correlations (*r*_MZ_ = 0.36–0.84) were over twice the DZ correlations (*r*_DZ_ = 0.00–0.44), suggesting dominant genetic effects. In parent-report ADHD scales, DZ variances were also greater than MZ variances, suggesting a potential negative sibling interaction (or contrast) effect (Rietveld et al., 2003). We therefore fitted ADE models with sibling interaction terms included in the parent-report ADHD symptoms (ADE-b).

#### Assessing the relationship between ADHD symptoms and emotional problems using direction of causation models

As is shown in Tables 2 and 3, during early childhood and mid-childhood, models with both a moderate positive genetic correlation (*r*g_EC_ = 0.52; *r*g_MC_ = 0.85) and a small negative causal effect (*b*_EC_ = −0.18; *b*_MC_ = −0.26) from ADHD symptoms to emotional problems provided the best fit to the data. From early adolescence onwards, models in which A and E components correlated with one another provided the same or similar fit to the data as models in which emotional problems causally influenced ADHD symptoms, with a difference in AIC of 1 or less. AIC weights did not come down heavily in favor of any one model. In causal models from early adolescence to early adulthood, causal effects running from emotional problems to ADHD symptoms were estimated as positive. As bivariate etiological correlation models involve fewer assumptions and no implications regarding causality, we select these models as the best fitting, while acknowledging that it may be the case that emotional problems causally influence ADHD from early adolescence onwards. In either model, the genetic covariance significantly contributed to the association between ADHD symptoms and emotional problems. In bivariate etiological correlation models, additive genetic correlations (*r*g = 0.62–0.66) explained a significant portion of the shared variance and correlations between nonshared environmental factors (*r*e = 0.10–0.14) explained the rest of the association.

**Table 2.** Fit statistics for bivariate etiological correlation models, direction of causation models, and hybrid causal-correlation models at each stage_overall ADHD and emotional problems

Model	−2LL	ep	AIC (weight)	df	diffLL	CFI	TFI	RMSEA	p
1 Early childhood									
1.1 Bivariate aetiological correlation model	382 118	46	382 210 (0.00)	107 378	NA	0.94	0.94	0.03	NA
1.2 ADHD to Emo	382 703	45	382 793 (0.00)	107 379	585.11	0.93	0.94	0.04	<0.001
1.3 Emo to ADHD	382 532	45	382 622 (0.00)	107 379	414.15	0.93	0.94	0.04	<0.001
**1.4 rA + ADHD to Emo**	**382 087**	**46**	**382 179 (1.00)**	**107 378**		0.94	0.94	0.03	
1.5 rA + Emo to ADHD	382 118	46	382 210 (0.00)	107 378		0.94	0.94	0.03	
2 Mid-childhood									
2.1 Bivariate aetiological correlation model	363 149	40	363 229 (0.00)	76 664	NA	0.96	0.96	0.03	NA
2.2 ADHD to Emo	363 596	39	363 674 (0.00)	76 665	447.24	0.95	0.95	0.03	<0.001
2.3 Emo to ADHD	363 569	39	363 647 (0.00)	76 665	420.48	0.95	0.95	0.03	<0.001
**2.4 rA + ADHD to Emo**	**363 089**	**40**	**363 169 (1.00)**	**76 664**		0.96	0.96	0.03	
2.5 rA + Emo to ADHD	363 155	40	363 235 (0.00)	76 664		0.96	0.96	0.03	
3 Early adolescence									
**3.1 Bivariate aetiological correlation model**	**356 322**	**39**	**356 400 (0.38)**	**79 633**	**NA**	**0.89**	**0.90**	**0.04**	**NA**
3.2 ADHD to Emo	356 398	38	356 474 (0.00)	79 634	76.17	0.89	0.90	0.04	<0.001
3.3 Emo to ADHD	356 612	38	356 688 (0.00)	79 634	290.36	0.88	0.90	0.04	<0.001
3.4 rA + ADHD to Emo	356 335	39	356 413 (0.01)	79 633		0.89	0.90	0.04	
3.5 rA + Emo to ADHD	356 321	39	356 399 (0.62)	79 633		0.89	0.90	0.04	
4 Late adolescence									
**4.1 Bivariate aetiological correlation model**	**328 033**	**39**	**328 111 (0.50)**	**69 142**	**NA**	**0.83**	**0.84**	**0.04**	**NA**
4.2 ADHD to Emo	328 068	38	328 144 (0.00)	69 143	34.91	0.82	0.84	0.04	<0.001
4.3 Emo to ADHD	328 140	38	328 217 (0.00)	69 143	107.27	0.82	0.84	0.05	<0.001
4.4 rA + ADHD to Emo	328 052	39	328 130 (0.00)	69 142		0.83	0.84	0.04	
4.5 rA + Emo to ADHD	328 033	39	328 111 (0.50)	69 142		0.83	0.85	0.04	
5 Early adulthood									
**5.1 Bivariate aetiological correlation model**	**379 004**	**43**	**379 090 (0.50)**	**74 369**	**NA**	**0.81**	**0.83**	**0.04**	**NA**
5.2 ADHD to Emo	379 115	42	379 199 (0.00)	74 370	110.38	0.80	0.82	0.04	<0.001
5.3 Emo to ADHD	379 238	42	379 322 (0.00)	74 370	233.25	0.80	0.82	0.04	<0.001
5.4 rA + ADHD to Emo	379 020	43	379 106 (0.00)	74 369		0.81	0.83	0.04	
5.5 rA + Emo to ADHD	379 004	43	379 090 (0.50)	74 369		0.81	0.83	0.04	

ep, estimated parameters of the comparison model; −2LL, minus 2*log-likelihood of the comparison model.

diffLL, difference in −2LL of the base and comparison models; df, degrees in freedom of the comparison model.

The models in bold type are the best-fitting models.

Bivariate etiological correlation models include the covariance between ADHD and emotional problems via genetic and environmental correlations (online Supplementary Fig. S1a); ADHD to Emo models are direction of causation models with effects running from ADHD to emotional problems (online Supplementary Fig. S1b); Emo to ADHD models are direction of causation models with effects running from emotional problems to ADHD (online Supplementary Fig. S1c); rA + ADHD to Emo models include both a genetic correlation and causation path running from ADHD to emotional problems (online Supplementary Fig. S1e); rA + Emo to ADHD models include a genetic correlation and causation path running from emotional problems to ADHD (online Supplementary Fig. S1f).

**Table 3.** Genetic effects, environmental effects, and causality in the association between emotional problems and ADHD subdomains from the best fitting models at the different timepoints

	rA	rE	Causality (ADHD to emo)	Causality (emo to ADHD)
Early childhood				
ADHD→Emo	0.52 (0.49–0.56)	–	−0.18 (−0.14 to −0.22)	–
Hyperactivity-impulsivity(I)→Emo	0.37 (0.33–0.42)	–	−0.14 (−0.11 to −0.18)	–
Inattention(H)-Emo	0.19 (0.16–0.22)	−0.03 (−0.06 to 0.00)	–	–
Mid-childhood				
ADHD→Emo	0.85 (0.82–0.88)	–	−0.26 (−0.31 to −0.21)	–
Hyperactivity-impulsivity(I)→Emo	0.81 (0.77–0.84)	–	−0.24 (−0.30 to −0.18)	–
Inattention(H)→Emo	0.76 (0.66–0.80)	–	−0.05 (−0.09 to −0.01)	–
Early adolescence				
ADHD-Emo	0.65 (0.58–0.75)	0.10 (0.07–0.13)	–	–
Hyperactivity-impulsivity(I)→Emo	0.76 (0.61–.81)	–	−0.07 (−0.13,−0.00)	–
Hyperactivity-impulsivity(I)-Emo	0.76 (0.55–0.81)	−0.01 (−0.06to 0.02)		
Inattention(H)→Emo	0.73 (0.57–0.78)	–	0.14 (0.09–0.19)	–
Late adolescence				
ADHD-Emo	0.66 (0.46–0.79)	0.14 (0.10–0.17)	–	–
Hyperactivity-impulsivity(I)-Emo	0.73 (0.37–0.78)	0.01 (−0.03–0.05)	–	–
Inattention(H)-Emo	0.76 (0.65–0.78)	0.11 (0.07–0.15)	–	–
Early adulthood				
ADHD-Emo	0.62 (0.58–0.67)	0.12 (0.09–0.15)	–	–
Hyperactivity-impulsivity(I)→Emo	0.33 (0.25–0.41)	–	−0.12(−0.18 to −0.06)	–
Hyperactivity-impulsivity(I)-Emo	0.25 (0.19–0.30)	−0.06 (−0.10 to −0.02)		
Inattention(H)→Emo	0.26 (0.16–0.35)	–	0.36 (0.30–0.41)	–

Hyperactivity(I)/inattention(H), results after controlling for the other subdomain.

Emo, emotional problems.

ADHD→Emo, causal models of ADHD on emotional problems.

Hyperactivity-impulsivity→Emo, causal models of hyperactivity-impulsivity on emotional problems.

Inattention→Emo, causal models of inattention on emotional problems.

ADHD-Emo, bivariate etiological correlation models of ADHD and emotional problems.

Hyperactivity-impulsivity-Emo, bivariate etiological correlation models of hyperactivity-impulsivity and emotional problems.

Inattention-Emo, bivariate etiological correlation models of inattention and emotional problems.

Brackets show 95% confidence intervals.

We next ran models to assess the association between emotional problems and each subdomain of ADHD while controlling for the other (see online Supplementary Tables S3 and S4). We found that the negative causal path from ADHD symptoms to emotional problems in childhood was mostly driven by hyperactivity-impulsivity. The models with both positive genetic correlations (*r*g_EC_ = 0.37; *r*g_MC_ = 0.81) and negative causal effects (*b*_EC_ = −0.14; *b*_MC_ = −0.24) from hyperactivity-impulsivity to emotional problems were the better fitting models during early childhood and mid-childhood, demonstrating the lowest AIC value and AIC weights of 1.00. From early adolescence, it became less clear which of our models was the best fitting model, but it was apparent that the association between hyperactivity and emotional problems was still driven by genetic overlap. In early adolescence and adulthood, models with positive genetic correlations (*r*g_EA_ = 0.76 and *r*g_A_ = 0.33) and small causal effects from hyperactivity-impulsivity to emotional problems (*b*_EA_ = −0.07; *b*_A_ = −0.12) had the lowest AIC values and the highest AIC weight. Models with both genetic correlations (*r*g_EA_ = 0.76; *r*g_A_ = 0.25) and very small environmental correlations (*r*e_EA_ = −0.01; *r*e_A_ = −0.06) had AIC scores that were only 3 and 4 larger than the causal models (in Table 3 we include results from both models). In late adolescence AIC scores and AIC weights did not distinguish between models with genetic correlations with causal effects and those with genetic correlations and environmental correlations. Because bivariate etiological correlation models involve fewer assumptions and no implications regarding causality, and because causal effects were estimated as very small, we present the bivariate etiological correlation models as the ‘best fitting’ in late adolescence. Taken together, our models suggests that the negative causal path from hyperactivity-impulsivity to emotional problems may persist in early adolescence and early adulthood, but the strength of the effect, and the evidence for it, appears diminished compared to that observed in childhood. It is noteworthy that these subdomain-specific effects in early adolescence and early adulthood were not detectable in the ADHD: emotional problems models.

For inattention and emotional problems, in early childhood it was difficult to distinguish a ‘best fitting’ model using AIC scores and weights. We therefore highlight the bivariate etiological correlation model as best fit, noting that parameter estimates suggest the association between inattention and emotional problems in early childhood is entirely attributable to genetic overlap. In mid-childhood, the model with the smallest AIC and largest AIC weight included a genetic correlation (*r*_MC_ = 0.76) and a small negative causal path (*b*_MC_ = −0.05) from inattention to emotional problems. In early adolescence and adulthood, the best fitting models included a genetic correlation (*r*_EA_ = 0.73; *r*_A_ = 0.26) and a positive causal influence of inattention on emotional problems (*b*_EA_ = 0.14; *b*_A_ = 0.36). In late adolescence there was no obvious best fitting model, so we highlight the bivariate aetiological correlation model as best fit, noting that the positive non-shared environment correlation is consistent with the positive causal effect of inattention on emotional problems found in early adolescence and early adulthood.

These results suggest that the negative causal effects from ADHD symptoms to emotional problems during early and middle childhood were largely driven by hyperactivity-impulsivity, with some evidence for positive causal effects of inattention on emotional problems from early adolescence. All models suggest that associations between ADHD and emotional problems are driven by genetic overlap.

### Sensitivity analysis

We found clear evidence for a negative causal effect of ADHD (hyperactivity-impulsivity) on emotional problems in early childhood and mid-childhood only. In early childhood and mid-childhood, we only had parent report measures of ADHD symptoms and emotional problems, self-report measures were available from early adolescence onwards. It is possible therefore that our childhood-specific findings were specific to parent report, rather than specific to childhood. To test this, we conducted sensitivity analyses in which we dropped all self-report scales from the adolescent models and repeated the model fitting process. Due to limited parental report information during early adulthood, our sensitivity analysis was restricted to adolescence. When only including the parent report scales during adolescence, most of our conclusions did not change, with one exception: in the model involving inattention and emotional problems in early adolescence, the positive causal effect from inattention to emotional problems diminished. In summary, our sensitivity analyses did not indicate that the inclusion of self-report symptoms explained the change in mechanism underlying associations between ADHD and emotional problems from early adolescence.

## Discussion

In the present study, we used the TEDS sample to explore the association between ADHD symptoms and emotional problems in a general population sample. In line with previous research (D'Agati et al., 2019; Sadek, 2016, 2018), we found that the correlation between ADHD symptoms and emotional problems increased from early childhood to early adulthood. In particular, the increase from mid-childhood to early adolescence was significant. Our analyses suggested that this was primarily driven by an increase in the association between inattention and emotional problems, the link between hyperactivity-impulsivity and emotional problems actually became weaker after early adolescence. These results are consistent with findings reported in previous studies which have shown that compared to hyperactivity-impulsivity, inattention is more likely to persist to later stages (Döpfner et al., 2015; Holbrook et al., 2016; Larsson et al., 2011), and is associated with increased problems within school and workplace settings (Merrell, Sayal, Tymms, & Kasim, 2017; Pope, 2010).

Moderate to high genetic correlations between ADHD symptoms and emotional problems were detected in all our models. Similar results have been reported in previous studies in independent samples (Chen et al., 2016; Rydell, Taylor, & Larsson, 2017; Stern et al., 2020), which highlighted the role genetic overlap plays in the association between the two traits from childhood to adulthood. The impact of nonshared environmental influences and causal relationships on covariance varied with age. In childhood, our best fitting models indicated that ADHD symptoms may act as a protective factor for the development of emotional problems. This was contrary to our expectations. When distinguishing ADHD subdomains, it became apparent that this protective effect was mostly driven by hyperactivity-impulsivity rather than inattention. Hyperactivity-impulsivity symptoms increase the activity level of children, and this may help to reduce emotional problems. For example, increased arousal and dopamine release associated with hyperactivity may contribute to less emotional problems (Dunlop & Nemeroff, 2007; Felger & Treadway, 2017). From adolescence, the persistence of ADHD is largely due to the continuity of inattention, with hyperactive symptoms decreasing as children grow older (Ramtekkar, Reiersen, Todorov, & Todd, 2010). This decrease in hyperactivity-impulsivity may lead to a decrease in the protective effect. Alternatively, the childhood-limited protective effect of hyperactivity-impulsivity could reflect increased academic and social challenges (Graber & Brooks-Gunn, 1996; Zarrett & Eccles, 2006). Hyperactivity-impulsivity is less likely to be adaptive when children grow older. For example, a previous study showed that the correlation between hyperactivity-impulsivity and peer negative nominations increases as children age from 9 to 13 years old (Pope, Bierman, & Mumma, 1989). It is possible hyperactive-impulsive behaviors lead to peer acceptance early in life, thus protecting against emotional problems, but as people age such behaviors become less accepted by peers. In addition, hyperactivity often accompanies mood dysregulation (Skirrow, McLoughlin, Kuntsi, & Asherson, 2009), which may mask emotional problems due to unstable mood state. Children with higher levels of ADHD symptoms are also more likely to receive treatment as well (Danielson, Visser, Chronis-Tuscano, & DuPaul, 2018), which could be beneficial to their mental health, thus reducing the link with emotional problems.

Causal paths from inattention to emotional problems became positive in adolescence and early adulthood. The differences between inattention and hyperactivity-impulsivity may be explained by their different manifestations. Inattention is not likely to mask emotional problems, on the contrary, it is even one of the symptoms of depression (Ciuhan & Iliescu, 2021). When children with ADHD symptoms, particularly inattention problems, transition into adolescence, they often face increased academic challenges and peer rejection (McKay, Kirk, Martin, & Cornish, 2023; Modesto-Lowe, Chaplin, Godsay, & Soovajian, 2014; Sayal, Washbrook, & Propper, 2015). These challenges may subsequently contribute to the development of emotional problems (Humphreys et al., 2013; Powell et al., 2020).

### Strengths and weaknesses of the current study

The current study is the first to investigate the association between ADHD symptoms and emotional problems from early childhood to early adulthood within a single sample. This allows us to investigate the correlation and the underlying mechanism across development. The co-occurrence of positive genetic overlap and negative phenotypic causality in the childhood models is one of the main novel findings in this study. It sheds light on why the two traits are associated with each other beyond their shared genetic influence.

Study limitations are as follows. First, the reporting of symptoms was limited to parent report scales when children were young (early childhood and mid-childhood). Externalizing problems are more observable than internalizing problems and the higher levels of externalizing problems may mask the internalizing symptoms when parents observe their children. Furthermore, parents may contrast their twin's ADHD symptoms, which may lead to an underestimation of symptoms in children with milder symptoms than their twin. Twin correlations for parent-reported ADHD symptoms were low in DZ twins. In response, we incorporated sibling contrast effects into our models to deal with this known bias when including parents reported ADHD. In addition, in order to test whether our childhood-specific findings were specific to parent report rather than specific to childhood, we conducted sensitivity analyses, showing that similar results were obtained when only including the parent report scales in adolescence and early adulthood. This indicated that our findings were not attributable to the use of self-report scales after childhood. Another potential limitation is that sample attrition meant that our sample became smaller with increasing age, which may have led to lower power to distinguish different models at later stages. However, even in early adulthood, the sample was still large enough (more than 5000 participants) to test different unidirectional causation models (Heath et al., 1993). In TEDS, as in most studies, attrition is related to mental health, such that individuals with more severe mental health issues tend to be absent from later assessments (Rimfeld et al., 2019; Teague et al., 2018). However, our analyses suggest that the magnitude of association(s) between (subdomains of) ADHD were unaffected by attrition. Re-estimating early childhood associations only in participants who remained in the study at later waves did not lead to noticeable changes in estimates. Similar findings have been reported in other studies (Gustavson, von Soest, Karevold, & Røysamb, 2012) and demonstrate that attrition can impact estimates of prevalence without impacting estimates of association. Additionally, there are only two items in the SDQ that assess inattention symptoms, and the Cronbach's alpha of some SDQ subscales is relatively low. These limitations in measurement might also influence the reliability of the assessment of ADHD subdomains and emotional symptoms. Better measurement tools need to be considered for future research on the comorbidity of ADHD symptoms and emotional problems. Finally, it is also worth noting that our study was conducted in a non-clinical sample, so while our findings give insight into the nature of links between emotional symptoms and ADHD symptoms, we cannot be certain that our findings will apply to children with diagnoses of ADHD. Future research should seek to test this.

## Conclusion

The present study demonstrated that in a general population sample, the correlations between ADHD symptoms and emotional problems increase from early childhood to early adulthood, with this increase largely driven by an increase in links between inattention and emotional problems, rather than between hyperactivity and emotional problems. At all developmental stages from early childhood to early adulthood the association between ADHD symptoms and emotional problems was significantly explained by shared genetic effects. During early development, hyperactivity-impulsivity symptoms appeared to either modestly protect children from developing emotional problems or to make the detection of emotional problems more difficult. After adolescence, inattention symptoms may contribute to the development of emotional problems. This study proposes new perspectives to understand the association between ADHD symptoms and emotional problems during different developmental stages.

## Supporting information

You et al. supplementary material You et al. supplementary material

## Supplementary material

The supplementary material for this article can be found at https://doi.org/10.1017/S0033291724002514
