# Supplementary material for: Exploring associations between ADHD symptoms and emotional problems from childhood to adulthood: shared aetiology or possible causal relationship?
Source: Psychol Med. 2024 Nov 18;54(15):4231–42. doi: 10.1017/S0033291724002514 (PMC11650170; doi:10.1017/S0033291724002514)
Supplement: You et al. supplementary material [file S0033291724002514sup001.docx]

**Supporting Information**

**Supplementary Table S1**. Correlations between childhood ADHD and emotional problems by only using the data from participants who were followed to later stages of the study

**Supplementary Table S2**. Means (SD) of raw scores for all the scales & correlations in monozygotic (MZ) and dizygotic (DZ) twins

**Supplementary Table S3**. Fit statistics of bivariate aetiological correlation model, direction of causation model and hybrid causal-correlation models at each stage_Hyperactivity-Impulsivity and Emotional problems (control Inattention)

**Supplementary Table S4**. Fit statistics of bivariate aetiological correlation model, direction of causation model and hybrid causal-correlation models at each stage_Inattention and Emotional problems (control Hyperactivity-Impulsivity)

**Supplementary Table S5**. Parameter estimates for emotional problems and ADHD (95% confidence intervals) in every stage from the best-fitting model per timepoint (control for the other subdomain)

**Supplementary Table S6**. Fit statistics of bivariate aetiological correlation model, direction of causation model and hybrid causal-correlation models in adolescence_ADHD and Emotional problems (only parent report scales)

**Supplementary Table S7**. Parameter estimates for emotional problems and ADHD (95% confidence intervals) in every stage from the best-fitting model per timepoint (only parent report scales)

**Supplementary Table S8**. Genetic effects, environmental effects and causality in the association between emotional problems and ADHD subdomains from the best models at the different timepoints (only parent report scales)

| **Supplementary Table S1.** Correlations between childhood ADHD and emotional problems by only using the data from participants who were followed to later stages of the study | | | | | |
| --- | --- | --- | --- | --- | --- |
| Correlations using complete sample | 2 to 4 years^1^ | 2 to 9 years^2^ | 2 to 12 years^3^ | 2 to 16 years^4^ | 2 to 21 years^5^ |
| Early childhood ADHD-emo | .30(.28,.32) | .29(.27,.32) | .28(.24,.32) | .31(.24,.38) | .32(.22,.41) |
| Early childhood inattention(H)-emo | .14(.12,.16) | .15(.11,.19) | .13(.08,.17) | .12(.07,.17) | .12(.04,.19) |
| Early childhood hyperactivity-impulsibity(I)-emo | .18(.16,.20) | .14(.11,.18) | .16(.12,.21) | .18(.13,.23) | .21(.14,.28) |

^1^Correlations between early-childhood ADHD and emotional problems by only using the data from participants who participated in the study from 2 to 4 years.

^2^Correlations between early-childhood ADHD and emotional problems by only using the data from participants who participated in the study from 2 to 9 years.

^3^Correlations between early-childhood ADHD and emotional problems by only using the data from participants who participated in the study from 2 to 12 years.

^4^Correlations between early-childhood ADHD and emotional problems by only using the data from participants who participated in the study from 2 to 16 years.

^5^Correlations between early-childhood ADHD and emotional problems by only using the data from participants who participated in the study from 2 to 21 years.

Brackets show 95% confidence intervals.

| **Supplementary Table S2**. Means (SD) of raw scores for all the scales & correlations in monozygotic (MZ) and dizygotic (DZ) twins | | | | | | |
| --- | --- | --- | --- | --- | --- | --- |
|  |  | Total | MZ | DZ | CorMZ | CorDZ |
|  |  | M(SD) | M(SD) | M(SD) |  |  |
| Early childhood emotional problems | BeharE2(p) | 3.51(1.97) | 3.37(1.89) | 3.58(2.00) | .55(.52,.58) | .23(.20,.26) |
|  | BeharE3(p) | 3.64(2.15) | 3.49(2.11) | 3.72(2.18) | .54(.50,.57) | .21(.18,.24) |
|  | BeharE4(p) | 1.74(1.66) | 1.59(1.59) | 1.81(1.69) | .54(.51,.57) | .26(.24,.29) |
|  | SDQE4(p) | 1.39(1.44) | 1.36(1.41) | 1.40(1.45) | .56(.53,.58) | .31(.28,.33) |
| Mid-childhood emotional problems | SDQE7(p) | 2.20(1.84) | 2.23(1.84) | 2.18(1.84) | .61(.59,.64) | .38(.36,.41) |
|  | anxiety7(p) | 8.79(5.34) | 8.87(5.33) | 8.74(5.35) | .71(.69,.73) | .46(.44,.49) |
|  | SDQE9(p) | 1.73(1.90) | 1.77(1.94) | 1.71(1.88) | .83(.81,.85) | .56(.53,.58) |
|  | anxiety9(p) | 3.30(2.76) | 3.29(2.80) | 3.30(2.73) | .66(.63,.69) | .41(.38,.45) |
| Early adolescence emotional problems | MFQ12(p) | 1.17(2.33) | 1.11(2.27) | 1.21(2.36) | .60(.57,.63) | .38(.36,.42) |
|  | MFQ12(p) | 2.31(3.33) | 2.31(3.34) | 2.31(3.33) | .41(.38,.45) | .28(.25,.31) |
|  | SDQE12(p) | 1.82(1.93) | 1.82(1.92) | 1.81(1.93) | .60(.56,.62) | .32(.29,.35) |
|  | SDQE12(p) | 2.19(2.06) | 2.18(2.06) | 2.19(2.05) | .40(.35,.42) | .20(.17,.23) |
| Late adolescence emotional problems | ARBQ16(p) | 3.60(4.19) | 3.75(4.35) | 3.51(4.09) | .65(.64,.69) | .44(.41,.47) |
|  | MFQ16(p) | 0.99(2.30) | 0.94(2.29) | 1.01(2.31) | .56(.51,.58) | .26(.23,.30) |
|  | MFQ16(c) | 3.64(4.43) | 3.52(4.43) | 3.70(4.43) | .40(.36,.44) | .21(.18,.25) |
|  | SDQE16(c) | 2.75(2.24) | 2.73(2.26) | 2.76(2.23) | .41(.37,.49) | .16(.12,.19) |
| Early adulthood emotional problems | GA21(c) | 7.31(7.43) | 7.30(7.48) | 7.31(7.40) | .35(.30,.39) | .12(.08,.16) |
|  | SDQE21(p) | 2.37(2.29) | 2.40(2.31) | 2.35(2.29) | .55(.52,.58) | .22(.19,.25) |
|  | SDQE21(c) | 3.60(2.69) | 3.66(2.67) | 3.56(2.71) | .34(.29,.38) | .18(.14,.22) |
|  | MFQ21(c) | 4.45(4.11) | 4.47(4.19) | 4.44(4.07) | .33(.28,.38) | .15(.11,.19) |
| Early childhood ADHD | BeharA2(p) | 2.96(1.98) | 2.98(1.89) | 2.95(2.02) | .66(.64,.69) | .17(.14,.20) |
|  | BeharA3(p) | 2.82(1.99) | 2.82(1.90) | 2.81(2.03) | .64(.61,.67) | .04(.01,.07) |
|  | BeharA4(p) | 2.62(1.94) | 2.64(1.86) | 2.61(1.99) | .59(.57,.62) | .01(-.02,.04) |
|  | SDQA4(p) | 3.99(2.33) | 4.07(2.19) | 3.95(2.40) | .59(.57,.62) | .01(-.02,.04) |
| Mid-childhood ADHD | SDQA7(p) | 3.59(2.55) | 3.60(2.48) | 3.59(2.59) | .58(.56,.61) | .00(-.03,.03) |
|  | Conners8(p) | 11.18(9.36) | 11.25(9.30) | 11.14(9.39) | .84(.83,.85) | .41(.38,.43) |
|  | SDQA9(p) | 3.25(2.39) | 3.30(2.32) | 3.23(2.43) | .73(.70,.75) | .12(.09,.16) |
| Early adolescence ADHD | SDQA12(p) | 2.83(2.27) | 2.79(2.18) | 2.86(2.32) | .73(.71,.75) | .23(.20,.26) |
|  | SDQA12(c) | 3.53(2.30) | 3.42(2.27) | 3.58(2.32) | .47(.44,.51) | .18(.15,.21) |
|  | Conners12(p) | 9.86(8.61) | 9.75(8.45) | 9.92(8.70) | .84(.83,.86) | .44(.41,.46) |
| Late adolescence ADHD | Conners16(p) | 6.81(7.42) | 6.18(6.88) | 7.16(7.70) | .77(.75,.79) | .43(.40,.45) |
|  | SDQA16(p) | 2.27(1.98) | 2.15(1.85) | 2.33(2.04) | .71(.69,.73) | .23(.20,.27) |
|  | SDQA16(c) | 3.57(2.31) | 3.36(2.25) | 3.68(2.34) | .41(.37,.45) | .13(.09,.16) |
| Early adulthood ADHD | SDQA21(p) | 1.94(1.99) | 1.75(1.81) | 2.04(2.07) | .58(.55,.61) | .18(.15,.21) |
|  | SDQA21(c) | 3.31(2.19) | 3.26(2.18) | 3.34(2.19) | .36(.31,.40) | .16(.12,.20) |
|  | Conners21(p) | 6.48(7.11) | 5.86(6.63) | 6.83(7.35) | .74(.72,.76) | .37(.34,.40) |
|  | Conners21(c) | 13.63(8.90) | 13.31(8.66) | 13.83(9.03) | .44(.40,.49) | .14(.10,.19) |

p=parent report scales; c=child report scales.

BeharE2, BeharE3, BeharE4: the Preschool Behavior Questionnaire (Behar) Anxious-Fearful symptoms scales collected at 2, 3, and 4 years old.

BeharA2, BeharA3, BeharA4: the Preschool Behavior Questionnaire (Behar) hyperactivity-Distractible scales collected at 2, 3, and 4 years old.

SDQE4, SDQE7, SDQE9, SDQ12, SDQE16, SDQE21: Strengths and difficulties questionnaire (SDQ) emotional problems scales collected at 4, 7, 9, 12, 16, and 21 years old.

SDQA4, SDQA7, SDQA9, SDQA12, SDQA16, SDQA21: Strengths and difficulties questionnaire (SDQ) Hyperactivity-Impulsivity/inattention scales collected at 4, 7, 9, 12, 16, and 21 years old.

anxiety7, anxiety9: DSM-IV criteria for anxiety disorders and depression collected at 7 and 9 years old.

MFQ12, MFQ16: Short Mood and Feeling Questionnaire collected at 12 and 16 years old.

Conners8, Conners12, Conners16, Conners21: Conners rating revised scale collected at 8, 12, 16 and 21 years old.

ARBQ16: Anxiety-Related Behaviors Questionnaire collected at 16 years old.

GA21: Severity measure for generalized anxiety disorder – adult (GA) collected at 21 years old.

Brackets show 95% confidence intervals.

| **Supplementary Table S3**. Fit statistics of Bivariate aetiological correlation model, direction of causation model and hybrid causal-correlation models at each stage_Hyperactivity-Impulsivity and Emotional problems (control Inattention) | | | | | | | | | |
| --- | --- | --- | --- | --- | --- | --- | --- | --- | --- |
| models | -2LL | ep | AIC (weight) | df | diffLL | CFI | TFI | RMSEA | p |
| 1 Early childhood |  |  |  |  |  |  |  |  |  |
| 1.1 Bivariate aetiological correlation model | 336500 | 46 | 336592 (.00) | 107254 | NA | 0.94 | 0.94 | 0.03 | NA |
| 1.2 Hyperactivity-Impulsivity(I) to Emo | 336765 | 45 | 336855 (.00) | 107255 | 265.7 | 0.93 | 0.94 | 0.03 | <.001 |
| 1.3 Emo to Hyperactivity(I) | 336673 | 45 | 336763 (.00) | 107255 | 173 | 0.93 | 0.94 | 0.03 | <.001 |
| **1.4 rA+Hyperactivity(I) to Emo** | **336474** | **46** | **336566 (1.00)** | **107254** |  | **0.94** | **0.94** | **0.03** |  |
| 1.5 rA+Emo to Hyperactivity(I) | 336500 | 46 | 336592 (.00) | 107254 |  | 0.94 | 0.94 | 0.03 |  |
| 2 Mid-childhood |  |  |  |  |  |  |  |  |  |
| 2.1 Bivariate aetiological correlation model | 322742 | 40 | 322822 (.00) | 76673 | NA | 0.96 | 0.96 | 0.02 | NA |
| 2.2 Hyperactivity-Impulsivity(I) to Emo | 322979 | 39 | 323058 (.00) | 76674 | 237.24 | 0.95 | 0.95 | 0.02 | <.001 |
| 2.3 Emo to Hyperactivity-Impulsivity(I) | 322948 | 39 | 323026 (.00) | 76674 | 205.62 | 0.95 | 0.95 | 0.02 | <.001 |
| **2.4 rA+Hyperactivity-Impulsivity(I) to Emo** | **322723** | **40** | **322803 (1.00)** | **76673** |  | **0.96** | **0.96** | **0.02** |  |
| 2.5 rA+Emo to Hyperactivity-Impulsivity(I) | 322759 | 40 | 322839 (.00) | 76673 |  | 0.95 | 0.96 | 0.02 |  |
| 3 Early adolescence |  |  |  |  |  |  |  |  |  |
| 3.1 Bivariate aetiological correlation model | 322260 | 39 | 322338 (.16) | 79600 | NA | 0.89 | 0.90 | 0.03 | NA |
| 3.2 Hyperactivity-Impulsivity(I) to Emo | 322328 | 38 | 322404 (.00) | 79601 | 67.13 | 0.88 | 0.89 | 0.03 | <.001 |
| 3.3 Emo to Hyperactivity-Impulsivity(I) | 322324 | 38 | 322400 (.00) | 79601 | 63.76 | 0.88 | 0.90 | 0.03 | <.001 |
| **3.4 rA+Hyperactivity-Impulsivity(I) to Emo** | **322257** | **39** | **322335 (.74)** | **79600** |  | **0.88** | **0.90** | **0.03** |  |
| 3.5 rA+Emo to Hyperactivity-Impulsivity(I) | 322261 | 39 | 322339 (.10) | 79600 |  | 0.88 | 0.90 | 0.03 |  |
| 4 Late adolescence |  |  |  |  |  |  |  |  |  |
| **4.1 Bivariate aetiological correlation model** | **292698** | **39** | **292776 (.33)** | **69081** | **NA** | **0.83** | **0.85** | **0.04** | **NA** |
| 4.2 Hyperactivity-Impulsivity(I) to Emo | 292713 | 38 | 293789 (.00) | 69082 | 15.69 | 0.83 | 0.85 | 0.04 | <.001 |
| 4.3 Emo to Hyperactivity-Impulsivity(I) | 292711 | 38 | 292787 (.00) | 69082 | 12.98 | 0.83 | 0.85 | 0.04 | <.001 |
| 4.4 rA+Hyperactivity-Impulsivity(I) to Emo | 292698 | 39 | 292776 (.33) | 69081 |  | 0.83 | 0.85 | 0.04 |  |
| 4.5 rA+Emo to Hyperactivity-Impulsivity(I) | 292698 | 39 | 292776 (.33) | 69081 |  | 0.83 | 0.85 | 0.04 |  |
| 5 Early adulthood |  |  |  |  |  |  |  |  |  |
| 5.1 Bivariate aetiological correlation model | 335398 | 43 | 335484 (.11) | 74300 | NA | 0.93 | 0.94 | 0.02 | NA |
| 5.2 Hyperactivity-Impulsivity(I) to Emo | 335450 | 42 | 335534 (.00) | 74301 | 51.5 | 0.93 | 0.93 | 0.02 | <.001 |
| 5.3 Emo to Hyperactivity-Impulsivity(I) | 335454 | 42 | 335538 (.00) | 74301 | 56 | 0.93 | 0.93 | 0.02 | <.001 |
| **5.4 rA+Hyperactivity-Impulsivity(I) to Emo** | **335394** | **43** | **335480 (.79)** | **74300** |  | **0.93** | **0.94** | **0.02** |  |
| 5.5 rA+Emo to Hyperactivity-Impulsivity(I) | 335398 | 43 | 335484 (.11) | 74300 |  | 0.93 | 0.94 | 0.02 |  |

ep: estimated parameters of the comparison model; -2LL: minus 2*log-likelihood of the comparison model.

diffLL: difference in -2LL of the base and comparison models; df: degrees in freedom of the comparison model.

The models in bold type are the best-fitting models.

Hyperactivity-Impulsivity(I): results after control inattention.

Bivariate aetiological correlation models include the covariance between hyperactivity-impulsivity and emotional problems via genetic and environmental correlations (see Figure S1a); Hyperactivity-Impulsivity to Emo models are direction of causation models with effects running from hyperactivity-impulsivity to emotional problems (Figure S1b); Emo to Hyperactivity-Impulsivity models are direction of causation models with effects running from emotional problems to hyperactivity-impulsivity (Figure S1c); rA+Hyperactivity-Impulsivity to Emo models include both genetic correlation and causation paths running from hyperactivity-impulsivity to emotional problems (Figure S1e); rA+Emo to Hyperactivity-Impulsivity models include both genetic correlation and causation paths running from emotional problems to hyperactivity-impulsivity (Figure S1f).

| **Supplementary Table S4**. Fit statistics of Bivariate aetiological correlation model, direction of causation model and hybrid causal-correlation models at each stage_Inattention and Emotional problems (control Hyperactivity-Impulsivity) | | | | | | | | | | |
| --- | --- | --- | --- | --- | --- | --- | --- | --- | --- | --- |
| models | -2LL | ep | AIC (weight) | df | diffLL | CFI | TFI | RMSEA | p |  |
| 1 Early childhood |  |  |  |  |  |  |  |  |  |  |
| **1.1 Bivariate aetiological correlation model** | **322074** | **46** | **322166 (.27)** | **107254** | **NA** | **0.93** | **0.93** | **0.03** | **NA** |  |
| 1.2 Inattention(H) to Emo | 322165 | 45 | 322255 (.00) | 107255 | 91.03 | 0.92 | 0.93 | 0.03 | <.001 |  |
| 1.3 Emo to Inattention(H) | 322118 | 45 | 322208 (.00) | 107255 | 43.76 | 0.92 | 0.93 | 0.03 | <.001 |  |
| 1.4 rA+Inattention(H) to Emo | 322073 | 46 | 322165 (.45) | 107254 |  | 0.93 | 0.93 | 0.03 |  |  |
| 1.5 rA+Emo to Inattention(H) | 322074 | 46 | 322166 (.27) | 107254 |  | 0.93 | 0.93 | 0.03 |  |  |
| 2 Mid-childhood |  |  |  |  |  |  |  |  |  |  |
| 2.1 Bivariate aetiological correlation model | 314373 | 40 | 314453 (.07) | 76673 | NA | 0.95 | 0.95 | 0.02 | NA |  |
| 2.2 Inattention(H) to Emo | 314442 | 39 | 314520 (.00) | 76674 | 69.6 | 0.95 | 0.95 | 0.02 | <.001 |  |
| 2.3 Emo to Inattention(H) | 314391 | 39 | 314469 (.00) | 76674 | 18.73 | 0.95 | 0.95 | 0.02 | <.001 |  |
| **2.4 rA+Inattention(H) to Emo** | **314368** | **40** | **314448 (.88)** | **76673** |  | **0.95** | **0.95** | **0.02** |  |  |
| 2.5 rA+Emo to Inattention(H) | 314374 | 40 | 314454 (.04) | 76673 |  | 0.95 | 0.95 | 0.02 |  |  |
| 3 Early adolescence |  |  |  |  |  |  |  |  |  |  |
| 3.1 Bivariate aetiological correlation model | 312824 | 39 | 312902 (.04) | 79600 | NA | 0.88 | 0.89 | 0.03 | NA |  |
| 3.2 Inattention(H) to Emo | 312876 | 38 | 312952 (.00) | 79601 | 52.97 | 0.87 | 0.89 | 0.03 | <.001 |  |
| 3.3 Emo to Inattention(H) | 312869 | 38 | 312945 (.00) | 79601 | 45.43 | 0.88 | 0.89 | 0.03 | <.001 |  |
| **3.4 rA+Inattention(H) to Emo** | **312818** | **39** | **312896 (.79)** | **79600** |  | **0.88** | **0.89** | **0.03** |  |  |
| 3.5 rA+Emo to Inattention(H) | 312821 | 39 | 312899 (.18) | 79600 |  | 0.88 | 0.89 | 0.03 |  |  |
| 4 Late adolescence |  |  |  |  |  |  |  |  |  |  |
| **4.1 Bivariate aetiological correlation model** | **290789** | **39** | **290867 (.35)** | **69081** | **NA** | **0.84** | **0.86** | **0.04** | **NA** |  |
| 4.2 Inattention(H) to Emo | 290822 | 38 | 290898 (.00) | 69082 | 33.62 | 0.84 | 0.86 | 0.04 | <.001 |  |
| 4.3 Emo to Inattention(H) | 290821 | 38 | 290897 (.00) | 69082 | 32.15 | 0.84 | 0.86 | 0.04 | <.001 |  |
| 4.4 rA+Inattention(H) to Emo | 290792 | 39 | 290870 (.08) | 69081 |  | 0.84 | 0.86 | 0.04 |  |  |
| 4.5 rA+Emo to Inattention(H) | 290788 | 39 | 290866 (.57) | 69081 |  | 0.84 | 0.86 | 0.04 |  |  |
| 5 Early adulthood |  |  |  |  |  |  |  |  |  |  |
| 5.1 Bivariate aetiological correlation model | 333358 | 43 | 333444 (.07) | 74300 | NA | 0.90 | 0.91 | 0.03 | NA |  |
| 5.2 Inattention(H) to Emo | 333378 | 42 | 333462 (.00) | 74301 | 19.8 | 0.90 | 0.91 | 0.03 | <.001 |  |
| 5.3 Emo to Inattention(H) | 333404 | 42 | 333488 (.00) | 74301 | 45.6 | 0.89 | 0.91 | 0.03 | <.001 |  |
| **5.4 rA+Inattention(H) to Emo** | **333353** | **43** | **333439 (.86)** | **74300** |  | **0.90** | **0.91** | **0.03** |  |  |
| 5.5 rA+Emo to Inattention(H) | 333358 | 43 | 333444 (.07) | 74300 |  | 0.90 | 0.91 | 0.03 |  |  |

ep: estimated parameters of the comparison model; -2LL: minus 2*log-likelihood of the comparison model.

diffLL: difference in -2LL of the base and comparison models; df: degrees in freedom of the comparison model.

The models in bold type are the best-fitting models.

Inattention(H): results after control hyperactivity-impulsivity.

Bivariate aetiological correlation models include the covariance between inattention and emotional problems via genetic and environmental correlations (see Figure S1a); Inattention to Emo models are direction of causation models with effects running from inattention to emotional problems (Figure S1b); Emo to Inattention models are direction of causation models with effects running from emotional problems to inattention (Figure S1c); rA+Inattention to Emo models include both genetic correlation and causation paths running from inattention to emotional problems (Figure S1e); rA+Emo to Inattention models include both genetic correlation and causation paths running from emotional problems to inattention (Figure S1f).

| **Supplementary Table S5.** Parameter estimates for emotional problems and ADHD (95% confidence intervals) in every stage from the best-fitting model per timepoint (control for the other subdomain) | | | | | | | |
| --- | --- | --- | --- | --- | --- | --- | --- |
|  | Models | a12 (ADHD) | d12 | e12 | a22 (Emo) | c22 | e22 |
| Early childhood | ADHD_Emo | .27(.24,.31) | .42(.38,.46) | .31(.29,.33) | .72(.70,.74) | .00(.00,.02) | .28(.26,.30) |
|  | Hyperactivity-Impulsivity(I)_Emo | .14(.11,.17) | .49(.45,.53) | .37(.35,.40) | .70(.63,.73) | .01(.00,.06) | .29(.27,.31) |
|  | Inattention(H)_Emo | .04(.03,.05) | .51(.47,.55) | .45(.42,.49) | .68(.61,.71) | .01(.00,.07) | .31(.29,.34) |
| Mid-childhood | ADHD_Emo | .39(.34,.44) | .44(.38,.48) | .18(.16,.19) | .73(.68,.77) | .06(.03,.10) | .21(.19,.23) |
|  | Hyperactivity-Impulsivity(I)_Emo | .30(.24,.36) | .52(.45,.58) | .18(.16,.21) | .65(.60,.70) | .13(.09,.18) | .22(.20,.24) |
|  | Inattention(H)_Emo | .07(.04,.10) | .61(.56,.65) | .33(.29,.36) | .57(.51,.64) | .16(.10,.22) | .26(.24,.28) |
| Early adolescence | ADHD_Emo | .52(.40,.64) | .31(.20,.43) | .17(.15,.18) | .63(.54,.67) | .00(.00,.08) | .36(.33,.40) |
|  | Hyperactivity-Impulsivity(I)_Emo | .13(.08,.20) | .65(.58,.71) | .22(.19,.25) | .57(.47,.66) | .06(.00,.14) | .37(.33,.41) |
|  | Inattention(H)_Emo | .09(.05,.14) | .61(.55,.66) | .30(.27,.34) | .53(.43,.63) | .06(.00,.15) | .40(.36,.44) |
| Late adolescence | ADHD_Emo | .38(.25,.54) | .42(.26,.55) | .20(.18,.22) | .59(.51,.63) | .00(.00,.06) | .41(.37,.44) |
|  | Hyperactivity-Impulsivity(I)_Emo | .03(.01,.09) | .70(.66,.74) | .28(.24,.31) | .53(.42,.60) | .03(.00,.12) | .44(.40,.48) |
|  | Inattention(H)_Emo | .10(.08,.14) | .61(.57,.65) | .29(.26,.32) | .57(.47,.61) | .00(.00,.08) | .43(.39,.47) |
| Early adulthood | ADHD_Emo | .39(.34,.46) | .34(.27,.40) | .27(.24,.29) | .48(.44,.52) | .00(.00,.00) | .52(.48,.56) |
|  | Hyperactivity-Impulsivity(I)_Emo | .11(.06,.17) | .55(.48,.61) | .34(.30,.38) | .50(.45,.54) | .00(.00,.00) | .50(.46,.55) |
|  | Inattention(H)_Emo | .07(.02,.13) | .56(.48,.62) | .38(.34,.42) | .39(.34,.44) | .00(.00,.00) | .61(.56,.66) |

Hyperactivity-Impulsivity(I)/Inattention(H): results after control another subdomain.

Emo, emotional problems.

ADHD_Emo: models of ADHD and emotional problems.

Hyperactivity-Impulsivity(I)_Emo: models of hyperactivity-impulsivity and emotional problems.

Inattention(H)_Emo: models of inattention and emotional problems.

Brackets show 95% confidence intervals.

| **Supplementary Table S6**. Fit statistics of Bivariate aetiological correlation model, direction of causation model and hybrid causal-correlation models in adolescence (only parent report scales) | | | | | | | | | |
| --- | --- | --- | --- | --- | --- | --- | --- | --- | --- |
| models | -2LL | ep | AIC (weight) | df | diffLL | CFI | TFI | RMSEA | p |
| 1 Early adolescence_ADHD |  |  |  |  |  |  |  |  |  |
| 1.1 Bivariate aetiological correlation model^a^ | 206436 | 27 | 206490 (.27) | 45521 | NA | 0.99 | 0.99 | 0.02 | NA |
| 1.2 ADHD to Emo | 206483 | 26 | 206535 (.00) | 45522 | 47 | 0.99 | 0.99 | 0.02 | <.001 |
| 1.3 Emo to ADHD | 206669 | 26 | 206721 (.00) | 45522 | 232.23 | 0.98 | 0.98 | 0.02 | <.001 |
| 1.4 rA+ADHD to Emo | 206446 | 27 | 206500 (.00) | 45521 |  | 0.99 | 0.99 | 0.02 |  |
| 1.5 rA+Emo to ADHD | 206434 | 27 | 206488 (.73) | 45521 |  | 0.99 | 0.99 | 0.02 |  |
| 2 Early adolescence_Hyperactivity-Impulsivity |  |  |  |  |  |  |  |  |  |
| 2.1 Bivariate aetiological correlation model^b^ | 182057 | 27 | 182111 (.01) | 45478 | NA | 0.99 | 0.99 | 0.01 | NA |
| 2.2 Hyperactivity-Impulsivity(I) to Emo | 182147 | 26 | 182199 (.00) | 45479 | 89.56 | 0.98 | 0.98 | 0.02 | <.001 |
| 2.3 Emo to Hyperactivity-Impulsivity(I) | 182151 | 26 | 182203 (.00) | 45479 | 94.32 | 0.98 | 0.98 | 0.02 | <.001 |
| 2.4 rA+Hyperactivity-Impulsivity(I) to Emo | 182048 | 27 | 182102 (.99) | 45478 |  | 0.99 | 0.99 | 0.01 |  |
| 2.5 rA+Emo to Hyperactivity-Impulsivity(I) | 182060 | 27 | 182114 (.00) | 45478 |  | 0.99 | 0.99 | 0.01 |  |
| 3 Early adolescence_Inattention |  |  |  |  |  |  |  |  |  |
| 3.1 Bivariate aetiological correlation model^c^ | 180433 | 27 | 180487 (.23) | 45478 | NA | 0.98 | 0.99 | 0.02 | NA |
| 3.2 Inattention(H) to Emo | 180455 | 26 | 180507 (.00) | 45479 | 22.36 | 0.98 | 0.99 | 0.02 | <.001 |
| 3.3 Emo to Inattention(H) | 180442 | 26 | 180494 (.01) | 45479 | 9.2 | 0.98 | 0.99 | 0.02 | <.001 |
| 3.4 rA+Inattention(H) to Emo | 180434 | 27 | 180488 (.14) | 45478 |  | 0.98 | 0.99 | 0.02 |  |
| 3.5 rA+Emo to Inattention(H) | 180431 | 27 | 180485 (.62) | 45478 |  | 0.96 | 0.99 | 0.02 |  |
| 4 Late adolescence_ADHD |  |  |  |  |  |  |  |  |  |
| 4.1 Bivariate aetiological correlation model | 190117 | 27 | 190171 (.50) | 39514 | NA | 0.98 | 0.98 | 0.02 | NA |
| 4.2 ADHD to Emo | 190187 | 26 | 190239 (.00) | 39515 | 69.78 | 0.97 | 0.98 | 0.02 | <.001 |
| 4.3 Emo to ADHD | 190332 | 26 | 190384 (.00) | 39515 | 214.56 | 0.97 | 0.97 | 0.03 | <.001 |
| 4.4 rA+ADHD to Emo | 190133 | 27 | 190187 (.00) | 39514 |  | 0.97 | 0.98 | 0.02 |  |
| 4.5 rA+Emo to ADHD | 190117 | 27 | 190171 (.50) | 39514 |  | 0.98 | 0.98 | 0.02 |  |
| 5 Late adolescence_Hyperactivity-Impulsivity |  |  |  |  |  |  |  |  |  |
| 5.1 Bivariate aetiological correlation model | 166397 | 27 | 166451 (.12) | 39514 | NA | 0.98 | 0.98 | 0.02 | NA |
| 5.2 Hyperactivity-Impulsivity(I) to Emo | 166491 | 26 | 166543 (.00) | 39515 | 93.83 | 0.97 | 0.98 | 0.02 | <.001 |
| 5.3 Emo to Hyperactivity-Impulsivity(I) | 166448 | 26 | 166500 (.00) | 39515 | 50.82 | 0.98 | 0.98 | 0.02 | <.001 |
| 5.4 rA+Hyperactivity-Impulsivity(I) to Emo | 166394 | 27 | 166448 (.55) | 39514 |  | 0.98 | 0.98 | 0.02 |  |
| 5.5 rA+Emo to Hyperactivity-Impulsivity(I) | 166395 | 27 | 166449 (.33) | 39514 |  | 0.98 | 0.98 | 0.02 |  |
| 6 Late adolescence_inattention |  |  |  |  |  |  |  |  |  |
| 6.1 Bivariate aetiological correlation model | 169478 | 27 | 169532 (.38) | 39514 | NA | 0.98 | 0.98 | 0.02 | NA |
| 6.2 Inattention(H) to Emo | 169504 | 26 | 169556 (.00) | 39515 | 25.64 | 0.98 | 0.98 | 0.02 | <.001 |
| 6.3 Emo to Inattention(H) | 169505 | 26 | 169557 (.00) | 39515 | 26.18 | 0.98 | 0.98 | 0.02 | <.001 |
| 6.4 rA+Inattention(H) to Emo | 169479 | 27 | 169533 (.23) | 39514 |  | 0.98 | 0.98 | 0.02 |  |
| 6.5 rA+Emo to Inattention(H) | 169478 | 27 | 169532 (.38) | 39514 |  | 0.98 | 0.98 | 0.02 |  |

ep: estimated parameters of the comparison model; -2LL: minus 2*log-likelihood of the comparison model.

diffLL: difference in -2LL of the base and comparison models; df: degrees in freedom of the comparison model.

Hyperactivity-Impulsivity(I)/Inattention(H): results after control another subdomain.

Bivariate aetiological correlation models^a/b/c^ include the covariance between ADHD/hyperactivity-impulsivity/inattention and emotional problems via genetic and environmental correlations (see Figure S1a); ADHD/Hyperactivity-Impulsivity/Inattention to Emo models are direction of causation models with effects running from ADHD/hyperactivity-impulsivity/inattention to emotional problems (Figure S1b); Emo to ADHD/Hyperactivity-Impulsivity/Inattention models are direction of causation models with effects running from emotional problems to ADHD/hyperactivity-impulsivity/inattention (Figure S1c); rA+ADHD/Hyperactivity-Impulsivity/Inattention to Emo models include both genetic correlation and causation paths running from ADHD/hyperactivity-impulsivity/inattention to emotional problems (Figure S1e); rA+Emo to ADHD/Hyperactivity-Impulsivity/Inattention models include both genetic correlation and causation paths running from emotional problems to ADHD/hyperactivity-impulsivity/inattention (Figure S1f).

| **Supplementary Table S7**. Parameter estimates for emotional problems and ADHD (95% confidence intervals) in every stage from the best-fitting model per timepoint (only parent report scales) | | | | | | | |
| --- | --- | --- | --- | --- | --- | --- | --- |
|  | Models | a1^2^ (ADHD) | d1^2^ | e1^2^ | a2^2^ (Emo) | c2^2^ | e2^2^ |
| Early adolescence | ADHD_Emo | .66(.55,.76) | .18(.08,.29) | .16(.15,.18) | .65(.54,.68) | .00(.00,.09) | .35(.32,.39) |
|  | Hyperactivity-Impulsivity(I)_Emo | .25(.17,.34) | .55(.26,.63) | .20(.17,.22) | .58(.48,.67) | .11(.04,.19) | .31(.27,.35) |
|  | Inattention(H)_Emo | .13(.10,.17) | .58(.53,.62) | .29(.26,.33) | .56(.46,.66) | .09(.01,.17) | .35(.31,.39) |
| Late adolescence | ADHD_Emo | .52(.39,.63) | .29(.17,.41) | .20(.18,.22) | .62(.59,.66) | .00(.00,.00) | .38(.34,.41) |
|  | Hyperactivity-Impulsivity(I)_Emo | .13(.09,.19) | .61(.54,.66) | .26(.23,.30) | .43(.34,.52) | .20(.12,.27) | .37(.34,.41) |
|  | Inattention(I)_Emo | .11(.09,.13) | .60(.56,.63) | .29(.26,.32) | .62(.54,.65) | .00(.00,.06) | .38(.35,.42) |
|  |  |  |  |  |  |  |  |
|  |  |  |  |  |  |  |  |

Hyperactivity-Impulsivity(I)/Inattention(H): results after control another subdomain.

Emo, emotional problems.

ADHD_Emo: models of ADHD and emotional problems.

Hyperactivity-Impulsivity(I)_Emo: models of hyperactivity-impulsivity and emotional problems.

Inattention(H)_Emo: models of inattention and emotional problems.

Brackets show 95% confidence intervals.

| **Supplementary Table S8**. Genetic effects, environmental effects and causality in the association between emotional problems and ADHD subdomains from the best models at the different timepoints (only parent report scales) | | | | | |
| --- | --- | --- | --- | --- | --- |
|  |  | rA | rE | Causality (ADHD to emo) | Causality (emo to ADHD) |
| Early adolescence | ADHD-Emo | .60(.55,.66) | .13(.09,.16) | - | - |
|  | Hyperactivity-Impulsivity(I)→Emo | .76(.65,.82) |  | -.16(-.24,-.09) | - |
|  | Inattention(H)-Emo | .75(.68,.81) | .16(.13,.20) | - | - |
| Late adolescence | ADHD-Emo | .67(.59,.77) | .12(.08,.15) | - | - |
|  | Hyperactivity-Impulsivity(I)-Emo | .65(.55,.00) | .00(-.03,.03) | - | - |
|  | Inattention(H)-Emo | .79(.69,.81) | .14(.11,.18) | - | - |

Hyperactivity-Impulsivity(I)/Inattention(H): results after control another subdomain.

Emo, emotional problems.

Hyperactivity-Impulsivity→Emo: causal models of hyperactivity-impulsivity on emotional problems.

ADHD-Emo: bivariate aetiological correlation models of ADHD and emotional problems.

Hyperactivity-Impulsivity(I)-Emo: bivariate aetiological correlation models of hyperactivity-impulsivity and emotional problems.

Inattention(H)-Emo: bivariate aetiological correlation models of inattention and emotional problems.

Brackets show 95% confidence intervals.
